# Acute Supramaximal Exercise Increases the Brain Oxygenation in Relation to Cognitive Workload

**DOI:** 10.3389/fnhum.2016.00174

**Published:** 2016-04-20

**Authors:** Cem Seref Bediz, Adile Oniz, Cagdas Guducu, Enise Ural Demirci, Hilmi Ogut, Erkan Gunay, Caner Cetinkaya, Murat Ozgoren

**Affiliations:** ^1^Department of Physiology, Faculty of Medicine, Dokuz Eylul UniversityIzmir, Turkey; ^2^Department of Biophysics, Faculty of Medicine, Dokuz Eylul UniversityIzmir, Turkey; ^3^School of Sport Sciences and Technology, Dokuz Eylul UniversityIzmir, Turkey

**Keywords:** PFC, human factor, fNIRS, cognition and exercise, N-back test

## Abstract

Single bout of exercise can improve the performance on cognitive tasks. However, cognitive responses may be controversial due to different type, intensity, and duration of exercise. In addition, the mechanism of the effect of acute exercise on brain is still unclear. This study was aimed to investigate the effects of supramaximal exercise on cognitive tasks by means of brain oxygenation monitoring. The brain oxygenation of Prefrontal cortex (PFC) was measured on 35 healthy male volunteers via functional near infrared spectroscopy (fNIRS) system. Subjects performed 2-Back test before and after the supramaximal exercise wingate anerobic test (WAnT) lasting 30-s on cycle ergometer. The PFC oxygenation change evaluation revealed that PFC oxygenation rise during post-exercise 2-Back task was considerably higher than those in pre-exercise 2-Back task. In order to describe the relationship between oxygenation change and exercise performance, subjects were divided into two groups as high performers (HP) and low performers (LP) according to their peak power values (PP) obtained from the supramaximal test. The oxy-hemoglobin (oxy-Hb) values were compared between pre- and post-exercise conditions within subjects and also between subjects according to peak power. When performers were compared, in the HP group, the oxy-Hb values in post-exercise 2-Back test were significantly higher than those in pre-exercise 2-Back test. HP had significantly higher post-exercise oxy-Hb change (Δ) than those of LP. In addition, PP of the total group were significantly correlated with Δoxy-Hb.The key findings of the present study revealed that acute supramaximal exercise has an impact on the brain oxygenation during a cognitive task. Also, the higher the anerobic PP describes the larger the oxy-Hb response in post-exercise cognitive task. The current study also demonstrated a significant correlation between peak power (exercise load) and post-exercise hemodynamic responses (oxy-, deoxy- and total-Hb). The magnitude of this impact might be related with the physical performance capacities of the individuals. This can become a valuable parameter for future studies on human factor.

## Introduction

Individuals usually feel a mental arousal and define an increase in their cognitive abilities after exercise. The relation between exercise and cognitive function has been investigated in several decades. It has been proposed that exercise durations, exercise intensity and the differences in cognitive tasks could cause some contradicting results in previous studies (Ando et al., 2011; Endo et al., 2013; Schmit et al., 2015). Despite the large variations of the reported results, there is meta-analytic evidence that describes significant beneficial effects of acute exercise on cognition in the general population (Lambourne and Tomporowski, 2010; McMorris and Hale, 2012). The positive effects of erobic exercise on various cognitive tasks have been reported mostly. However there are rarely any reports about the effect of anerobic exercise (*i.e., the protocol in the current study*), characterized as short term and highly intensive effort, on cognition. The specific mechanisms by which exercise affects cognitive functions remain largely unclear (Rupp and Perrey, 2008). One of the mechanisms proposed is the relationship between prefrontal cortex (PFC) oxygenation and cognitive function. With the development of imaging methods, it has been possible to show the brain oxygenation during or post exercise conditions. Most of the studies demonstrate an increment in oxygenation of PFC following exercise (Rupp and Perrey, 2008; Jung et al., 2015).

In the literature, effects of exercise on cognitive performance were investigated during exercise and after exercise period. It is largely accepted that the metabolic activity in brain increases during cognitive tasks. The consumption of energy in the neurons depending on the metabolic activity increase leads an increase in the cerebral blood flow to meet the increased demand of oxygen and glucose. For this reason, the increment in brain oxygenation is accepted to be/as the physiological indicator for cognitive workload. Near infrared spectroscopy (NIRS) is a non-invasive measurement based sensitive method that indicates cerebral hemodynamic response (Ozgoren et al., 2012) to cognitive tasks by means of changing levels of oxy- and deoxy-HB (Albinet et al., 2014). Some studies reported a relationship between cerebral oxygenation level and cognitive test scores (Ayaz et al., 2007, 2012; McMorris et al., 2011; McMorris and Hale, 2012). Additionally, Li et al. (2005) and Gateau et al. (2015), describe a higher dorsolateral PFC activation during working memory task by using functional near infrared spectroscopy (fNIRS). Inadequate increase in cerebral oxygenation during cognitive task defined as an indicator of cerebral fatigue (Nybo and Rasmussen, 2007; Mandrick et al., 2013; Mehta and Parasuraman, 2014). Dietrich (2003) reported that, aside from cerebral fatigue, the hypo-frontality may occur due to the challenge of source allocation between the areas responsible for physical and cognitive workload.

We hypothesized that the brain oxygenation during cognitive task after the acute supramaximal exercise is higher than the pre-exercise cognitive task. We also expected a higher behavioral performance after the acute supramaximal exercise. This study aimed to evaluate the hemodynamic and behavioral changes of cognitive processes following an acute (anerobic) supramaximal exercise.

## Materials and Methods

### Participants and Experimental Design

**Figure 1:**
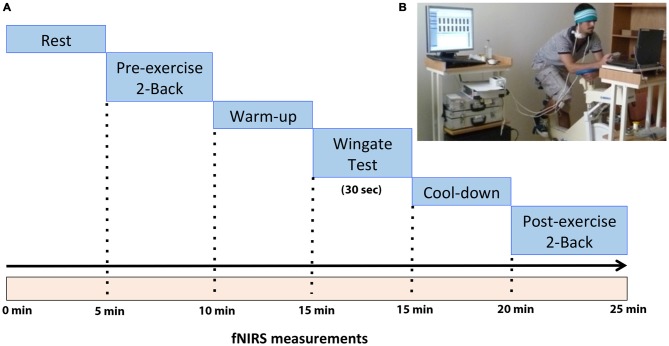
**(A)** The demonstration of experimental design for the current study. **(B)** Participant seated at cycle ergometer during whole paradigm.

Thirty-five male healthy and physically active subjects (1.78 ± 0.07 cm height and 72.1 ± 10.3 kg weight) aged between 18 and 23 years participated to the study (Table 1). All subjects were informed about the procedures and signed a written consent. Experiments were conducted in two visits. In first visit, the subjects were informed and became familiar to both exercise and N-Back test protocols. Within 3 days, subjects re-visited the laboratory to perform a short term-supramaximal acute exercise and cognitive tasks (2-Back tests) before and after exercise (Figure 1). Brain oxygenation was continuously measured via fNIRS during cognitive tasks and exercise. In order to evaluate the performance dependent results, an average peak power value was calculated for all subjects. Subjects who had higher power values than the average (751 Watt) was considered as high performers (HP), (*N* = 17 subjects) while who had lower power values than average were considered as low performers (LP), (*N* = 18 subjects). There were no statistically significant differences in terms of age, weight and height between the HP and LP groups. The Ethics Committee of Dokuz Eylul University approved all procedures and experimental design.

**Table 1:** ** Ages, heights, body weights, peak powers and maximal heart rates (Max-HR) of low performers (LP) and high performers (HP) groups were presented as mean ± SD**.

	Age (yrs)	Height (m)	Weight (kg)	Peak Power (Watts)	Max-HR (beats/min)
HP	21.0 ± 2.6	165.9 ± 22.5	72.7 ± 9.6	800.7 ± 77.3	165.4 ± 22.8
(*n* = 18)
LP	20.5 ± 2.1	168.9 ± 11.5	71.4 ± 11.1	634.3 ± 89.3	168.9 ± 11.5
(*n* = 17)

### Cognitive Task Procedure

Cognitive performance was evaluated by N-Back test before and after exercise. N-Back test, which mainly evaluates the working memory as well as sustained attention, was administrated to evaluate working memory and inhibitory control of irrelevant information (Jonides et al., 1997; Jaeggi et al., 2010). N-Back test was developed in OpenGL by using C programming language, has been administered to the participants by a laptop. A pseudorandom sequence of 120 letters consisting “K, Q, H, X, M, F, R, and B” were displayed on the center of the screen, one by one. Participants were asked to press the button on a response pad only if a letter on the screen was the same as the letter shown “*n*” steps earlier. In the present study “2-Back” condition was employed. The probability of each letter to be same as the two-step earlier letter was 30%. Each letter was presented on the computer screen for 500 ms and the inter-stimulus interval was set to vary between 1500 and 2000 ms. All tests were completed within 5 min. A brief training session, including a practice sequence of 15 letters, was given to all participants before the actual test to familiarize the subjects to the protocols. In order to evaluate working memory; reaction time for all stimuli total reaction time (TRT) and reaction time for the correct answers (CART), number of correct answers (CA), wrong answers (WA), and missed answers (M) have been obtained and measured. Nexus-10 bio-amplifier, which was equipped with appropriate sensors, was used to monitor the appearance of stimuli on the screen and participant responses in order to measure exact reaction times.

### Exercise Procedure

Wingate Anerobic test (WAnT) protocol was applied as the supramaximal exercise. WAnT is an exhaustive exercise for assessment of anerobic performance (Dotan and Bar-Or, 1983). WAnT is also accepted as a standardized anerobic exercise model for evaluating physiological responses to supramaximal exercise under controlled conditions (Weinstein et al., 1998). Following a warm up on the bicycle for 5 min at intensity about 50 watts, all subjects performed WAnT on a mechanically braked cycle ergometer with an optical pedal counter (Monark 824E, Sweden). Two unloaded 5-s sprints were performed during warm up. Following the warm up, subjects were instructed to pedal as fast as possible for 30 s against a resistance of 80 g/kg body mass. The subjects were verbally encouraged to maintain high pedaling rate throughout the WAnT. Pedal revolutions were monitored and recorded at 1-s intervals. Power outputs were calculated as described in Weinstein et al. (1998). The highest power output in the first 5 s of the test was used to represent the peak power (PP; PP = Distance × Load/ Time).

### fNIRS Recordings

The continuous wave (CW) fNIR system used in the present study (Imager 100, fNIR Devices LLC, MD, USA). System was connected to a flexible sensor pad, Sensor pad contained four light sources with built in peak wavelengths at 730 nm and 850 nm and 10 detectors designed to scan cortical areas underlying the forehead. The forehead area was cleaned with alcohol swap and scrubbing cream (NuPrep, USA). After the cleansing process the sensor was placed to the forehead region and covered by an elastic bandage specifically designed to hold it tightly across the head. Furthermore, a black head bandage was placed on top to eliminate the possible ambient light effects.

This system records two wavelengths and dark current for each of the 16 voxels, totaling 48 measurements for each sampling period (Ayaz et al., 2011, 2013). With a fixed source-detector separation of 2.5 cm, this configuration generates a total of 16 measurement locations (voxels) per wavelength. Data acquisition and visualization were conducted using COBI Studio software (Ayaz and Onaral, 2005). The fNIR device calculates relative changes to baseline values of oxy-hemoglobin (oxy-Hb) and deoxyhemoglobin (deoxy-Hb) molecules by means of a CW spectroscopy system that applies light to tissue at constant amplitude. The mathematical basis of CW-type measurements uses the modified Beer Lambert Law (Cope and Delpy, 1988). In the present study baseline condition was started at the beginning of the each task and lasted 20 s. The summation of oxy-Hb and deoxy-Hb values was described as total-Hb. Moreover, Δoxy-Hb and Δdeoxy-Hb parameters were used to describe the value differences (oxygenation change; i.e., rise or fall) between average values of two different sessions (i.e. Δ*OxyHb* = *OxyHb*_post_ − *OxyHb*_pre_) for both oxy-Hb and deoxy-Hb.

### fNIRS Analysis

**Figure 2:**
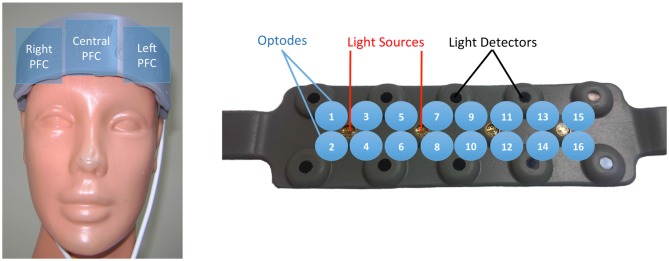
**The demonstration of functional near infrared spectroscopy (fNIRS) sensor pad.** Brain oxygenation was measured from 16 locations over the forehead area via fNIRS sensor (left side). The sensor pad has 4 light sources, 10 light detectors and 16 optodes (right side). Optodes that are located on the leftmost side of the forehead namely 1, 2, 3 and 4 denotes left prefrontal cortex (PFC), optodes that are centrally located as 7, 8, 9 and 10 denotes central PFC, and optodes that are located on the rightmost side as 13, 14, 15 and 16 denotes right PFC.

The raw intensity measurements at 730, and 850 nm were Butterworth low-pass filtered with MATLAB program (MATLAB and Statistics Toolbox, 2007). Butterworth filter was designed to eliminate possible respiration and heart rate signals and unwanted high frequency noise (Huppert et al., 2009). The artifact removal process has been made according to Ayaz et al. (2012). The PFC oxygenation data retrieved via fNIRS were examined in right, left and central PFC areas and defined as region of interest (ROI; Figure 2). Optodes that are located on the leftmost side of the forehead namely 1, 2, 3 and 4 combined to denote left PFC, optodes that are centrally located as 7, 8, 9 and 10 combined to denote central PFC, and optodes that are located on the rightmost side as 13, 14, 15 and 16 combined to denote right PFC. fNIRS signals were calculated and averaged over the whole pre- and post-exercise 2-Back sessions (Endo et al., 2013).

### Statistical Analysis

Shapiro-Wilk and Kolmogorov-Smirnov tests were used to control the normality of the data. All of the data were distributed normally. Age, height and weight differences between groups were tested via independent groups *t*-test, and the results were given in Table 1. We performed statistical analysis on combined channels as ROI wise.

A two-way group (LP vs. HP) and time (pre vs. post exercise) analysis of variance (ANOVA) was performed on fNIRS data and behavioral data. Moreover, oxy- and deoxy-Hb change were calculated as mentioned before (i.e., ΔOxyHb), and used for the statistical analysis. An independent group *t*-test was performed to test group differences (LP vs. HP) on oxy-, deoxy- and total-Hb changes. Also, the correlations between peak power and oxy-Hb, deoxy-Hb, and total-Hb changes were investigated via Spearman’s rank order correlations test.

## Results

### fNIRS Findings

**Figure 3:**
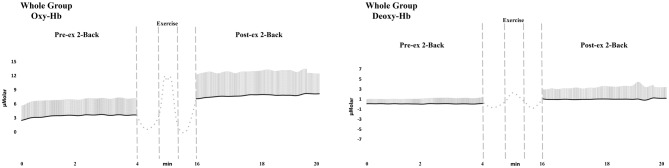
**The demonstration of oxy- and deoxy-Hb average levels in central PFC area during pre- and post-exercise 2-Back tests for the whole group.** Standard deviations are also marked in line with average as vertical lines (Note that only positive deflection is displayed for the sake of simplicity). Left panel indicated oxy-Hb and right panel indicated deoxy-Hb results. Vertical scale denotes the strength of fNIRS signal in μMolar units, which is normalized to baseline. Horizontal scale denotes the time scale in minutes. Vertical dashed lines denote pre-, during and post- exercise onsets and durations. The dotted lines represent the periods of warm-up and cool-down.

In the present study, 35 healthy subjects were recruited. In order to evaluate the performance dependent results, pre and post exercise PFC oxygenation levels during 2-Back tests were compared for both HP and LP groups with two-way mixed ANOVA. The oxy-Hb, deoxy-Hb, and total-Hb values of the groups were demonstrated for pre- and post-exercise 2-Back sessions were demonstrated in Table 2. Also the demonstration of oxy-Hb and deoxy-Hb levels in central PFC area during pre- and post-exercise 2-Back tests were given for whole group averages in Figure 3. All of the figures represent the group averages during related task (pre- and post-exercise 2-back).

**Table 2:** **Pre- and post-exercise 2-back oxy-, deoxy, and total-Hb levels (mean ± SD) are given for total, LP and HP group in central prefrontal cortex (PFC) area**.

	Pre-exercise 2-Back			Post-exercise 2-Back		
	Oxy-Hb (μMolar)	Deoxy-Hb (μMolar)	Total-Hb (μMolar)	Oxy-Hb (μMolar)	Deoxy-Hb (μMolar)	Total-Hb (μMolar)
Total Group	3.11 (± 3.41)	− 0.99 (± 0.94)	3.01 (± 3.71)	7.32 (± 4.85)***	0.84 (± 2.42)*	8.15 (± 6.56)***
HP	3.30 (± 3.62)	0.11 (± 0.94)	3.42 (± 4.04)	9.18 (± 4.90)***	1.84 (± 2.74)*	11.03 (± 6.88)***
LP	2.92 (± 3.30)	−0.30 (± 0.93)	2.62 (± 3.44)	5.54 (± 3.30)***	−0.11 (± 1.64)	5.43 (± 5.06)**

The significant differences related to comparisons of oxygenation, deoxygenation and total-Hb between pre- and post-exercise 2-Back session are marked with “*” (where “*” denotes p < 0.05, “**” denotes p < 0.01, and “***” denotes p < 0.001).

For oxygenation related analysis; pre- and post-exercise 2-Back tests oxy-Hb values were selected as within-subject factor and peak power was selected as between-subject factor. According to these analyses, oxygenation in post-exercise 2-Back test was higher than the oxygenation in pre-exercise 2-Back test for all three ROI of PFC [*F*_(1,33)_ = 51.82, *p* < 0.001, *η* = 0.61 for left; *F*_(1,33)_ = 78.42, *p* < 0.001, *η* = 0.70 for central; *F*_(1,33)_ = 54.25, *p* < 0.001 *η* = 0.61 for right]. For right PFC area there was not any interaction so, the pairwise comparisons were used. According to pairwise comparisons there was not any significant difference between groups, but there were significant differences between pre and post oxygenation levels in right PFC (*p* < 0.001). Because of the significant interaction in left and central PFC areas between within group effects (Pre/Post) and between group effects (HP/LP) in terms of two-way ANOVA results, between group analyses could not be evaluated (McDonald, 2009). Further analyses were made to clarify the statistical significance via within group paired samples *t*-test analysis. Paired samples *t*-test was conducted separately for each group and post-exercise 2-Back test oxy-Hb levels were found significantly higher than the pre-exercise 2-Back test oxy-Hb level for both group [for HP group: in left PFC, *T*_(16)_ = −5.99, *p* < 0.001; in central PFC, *T*_(16)_ = −8.12, *p* < 0.001; for LP group: in left PFC, *T*_(17)_ = −3.89, *p* < 0.001; in central PFC, *T*_(17)_ = −4.13, *p* < 0.001].

Moreover, deoxy-Hb related analyses were conducted. For the analyses; pre- and post-exercise 2-Back tests deoxy-Hb values were selected as within-subject factor and peak power was selected as between-subject factor. According to these analyses, deoxy-Hb values were significantly higher in post-exercise 2-Back test than the deoxy-Hb values in pre-exercise 2-Back test for all three ROI of PFC [*F*_(1,33)_ = 9.56, *p* < 0.01, *η* = 0.23 for left PFC; *F*_(1,33)_ = 7.03, *p* < 0.05, *η* = 0.17 for central PFC; *F*_(1,33)_ = 11.59, *p* < 0.01 *η* = 0.26 for right PFC]. For right and left PFC areas there was not any interaction so, the pairwise comparisons were used. According to pairwise comparisons there was not any significant difference between groups, but there were significant differences between pre and post deoxygenation levels in left and right PFC (*p* < 0.01 for both). Because of the significant interaction in central PFC area between within group effects (Pre/Post) and between group effects (HP/LP) in terms of two-way ANOVA results, between group analyses could not be evaluated (McDonald, 2009). Further analysis was made to clarify the statistical significance via within group paired samples *t*-test analysis. Paired samples *t*-test was conducted separately for each group and post-exercise 2-Back test deoxy-Hb levels were found significantly higher than the pre-exercise 2-Back test deoxy-Hb level for HP group in central PFC area [*T*_(16)_ = −2.67, *p* < 0.05] and there was not any significance for LP group in central PFC area.

Also total-Hb was significantly higher in post-exercise 2-Back test for all three interested ROI of PFC [*F*_(1,33)_ = 38.55, *p* < 0.001, *η* = 0.54 for left PFC; *F*_(1,33)_ = 47.20, *p* < 0.001, *η* = 0.59 for central PFC; *F*_(1,33)_ = 42.15, *p* < 0.001 *η* = 0.56 for right PFC]. For right and left PFC areas there was not any interaction so, the pairwise comparisons were used. According to pairwise comparisons there was not any significant difference between groups, but there were significant differences between pre and post total-Hb levels in left and right PFC (*p* < 0.001 for both). Because of the significant interaction in central PFC area between within group effects (Pre/Post) and between group effects (HP/LP) in terms of two-way ANOVA results, between group analyses could not be evaluated (McDonald, 2009). Further analyses were made to clarify the statistical significance via within group paired samples *t*-test analysis. Paired samples *t*-test was conducted separately for each group and post-exercise 2-Back test total-Hb levels were found significantly higher than the pre-exercise 2-Back test total-Hb level for HP group [in central PFC, *T*_(16)_ = −6.24, *p* < 0.001], and for LP group [in central PFC, *T*_(17)_ = −3.06, *p* < 0.01].

**Figure 4:**
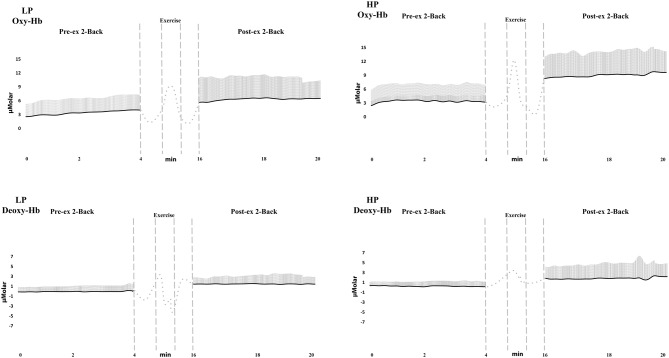
**The demonstration of oxy- and deoxy-Hb levels in central PFC area during pre- and post-exercise 2-Back tests for group averages.** Standard deviations are also marked in line with average as vertical lines (Note that only positive deflection is displayed for the sake of simplicity). The four panels are divided into performers (low and high performance, left and right consequently) and oxy- and deoxy-Hb (upper and lower consequently). Left panel indicated oxy-Hb and right panel deoxy-Hb results. Vertical scale denotes the strength of fNIRS signal in μMolar units, which is normalized to baseline. Horizontal scale denotes the time scale in minutes Vertical dashed lines denote pre-, during and post- exercise onsets and durations. The dotted lines represent the periods of warm-up and cool-down.

**Figure 5:**
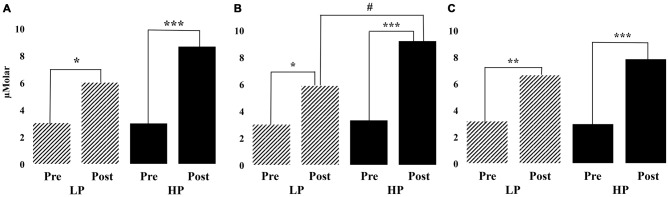
**The oxygenation values for pre- and post-exercise 2-Back sessions in left (A), center (B) and right (C) areas of PFC in high and low performance groups.** Within group significances are marked with “*” (where “*” denotes *p* < 0.05, “**” denotes *p* < 0.01, and “***” denotes *p* < 0.001); between group significances are marked with “^#^” (where “^#^” denotes *p* < 0.05).

In addition to these findings, the changes of oxy-Hb, deoxy-Hb, and total-Hb were compared between HP and LP groups via independent samples *t*-test (Table 3). While comparing the oxygenation changes (Δoxy-Hb) of the LP and HP group, PFC oxygenation rise in central PFC (*p* < 0.01) and left PFC (*p* < 0.05) areas were found significantly higher in HP group. While comparing the deoxygenation changes (Δdeoxy-Hb) of pre- and post-exercise 2-Back tests, HP group’s PFC deoxygenation changes in central PFC area was found significantly higher (*p* < 0.05). While comparing the total-Hb changes (Δtotal-Hb) of pre- and post-exercise 2-Back tests, HP group’s PFC total-Hb change in central PFC (*p* < 0.01) and left PFC (*p* < 0.05) areas were found significantly higher. Moreover, the peak power values (PP) of the total group were significantly correlated with Δoxy-Hb, Δdeoxy-Hb, and Δtotal-Hb values for central PFC area (*r* = 0.042 *p* < 0.01; *r* = 0.035 *p* < 0.02; *r* = 0.040 *p* < 0.01, respectively). The demonstration of average oxy-Hb and deoxy-Hb levels in central PFC area during pre- and post-exercise 2-Back Tests for HP and LP group were given in Figures 4, 5.

**Table 3:** **Post- and pre-exercise oxy-Hb, deoxy-Hb, and total-Hb changes (Δoxy-Hb, Δdeoxy-Hb, and Δtotal-Hb) are given for LP and HP groups in all PFC area (mean ± SD)**.

	ΔOxy-Hb (μMolar)			ΔDeoxy-Hb (μMolar)			ΔTotal-Hb (μMolar)		
	Left	Central	Right	Left	Central	Right	Left	Central	Right
HP	5.66 (± 3.89)*	5.88 (± 2.98)**	4.87 (± 3.48)	1.89 (± 1.91)	2.32 (± 2.13)*	1.26 (± 1.18)	7.10 (± 5.59)*	7.61 (± 5.03)**	5.74 (± 4.52)
LP	2.71 (± 2.94)	2.62 (± 2.69)	3.25 (± 3.48)	1.62 (± 1.67)	1.16 (± 0.89)	1.65 (± 1.56)	3.60 (± 4.58)	2.81 (± 3.90)	4.42 (± 4.72)

Significances between groups are marked with “*” (where “*” denotes p < 0.05, “**” denotes p < 0.01).

### Behavioral Findings

All subjects have successfully completed the whole procedure. 2-Back test scores (CA, WA, M, CART, TRT) were compared for total group and there were no significant differences between pre- and post-exercise 2-Back test scores. The same comparison was made for HP and LP groups and also there were no significant differences within (pre-exercise vs. post-exercise sessions) and between groups (Table 4). Though not statistically significant the reaction times, the correct answer CART were slightly progressed after the exercise.

**Table 4:** **The demonstration of correct answers, wrong answers, missed answers; correct answers reaction time, and total reaction time (mean ± SD) for high (HP) and low performers (LP) group during pre- and post-exercise 2-Back sessions**.

	Pre exercise 2-Back Session					Post exercise 2-Back Session				
	Correct answers	Wrong answers	Missed answers	Correct answers reaction time (ms)	Total reaction time (ms)	Correct answers	Wrong answers	Missed answers	Correct answers reaction time (ms)	Total reaction time (ms)
Total Group	25.49 (±5.65)	6.37 (±4.56)	10.34 (±5.57)	582.90 (±143.03)	596.60 (±147.39)	25.82 (±6.56)	7.97 (±4.61)	10.17 (±6.56)	576.60 (±186.63)	578.90 (±175.68)
HP	26.0 (±4.23)	6.12 (±4.21)	10.0 (±4.23)	561.76 (±148.61)	575.88 (±154.12)	26.29 (±6.12)	8.12 (±4.61)	9.71 (±6.12)	544.11 (±192.66)	551.17 (±181.71)
LP	25.0 (±6.56)	6.61 (±4.73)	10.67 (±6.45)	602.78 (±130.25)	616.11 (±133.46)	25.39 (±6.75)	7.83 (±4.48)	10.61 (±6.75)	607.22 (±169.70)	605.0 (±160.32)

## Discussion

Both physical benefits and cognitive improvements of the exercise have been frequently studied in the field. In the present study, the effects of anerobic exercise on brain oxygenation were investigated in relation to cognitive workload. The results revealed an increment in oxy-, deoxy-, and total-Hb levels on the post-exercise session of cognitive task. The increments in these parameters have been found to be statistically higher than the pre-exercise session of the cognitive task. In the related literature, it has been demonstrated that the oxy-Hb level increases in PFC during both physical exercise and cognitive task. Such rise has been linked to the increase in neural-metabolic activation (González-Alonso et al., 2004; Shibuya et al., 2004; Rupp and Perrey, 2008; Endo et al., 2013). In their study Tam and Zouridakis (2014) describes the oxy-Hb, deoxy-Hb, their summation (oxy-Hb + deoxy-Hb), and their difference (oxy-Hb—deoxy-Hb) measurements corresponding to the changes in oxygen delivery, oxygen extraction, total blood volume delivered, and total oxygenation, respectively. The increasing level of oxy- and deoxy-Hb is accepted as an indicator of an increase in blood flow (Endo et al., 2013). The present study revealed a rise in all oxy-Hb, deoxy-Hb, and total-Hb levels during both pre- and post-exercise cognitive tasks, which might be related to regional increase of cerebral blood flow. In this context, regional blood flow in PFC during post-exercise session of cognitive task could be considered higher than the pre-exercise session of cognitive task. Such increased hemodynamic responses may be an indicator for the additional effort during post-exercise cognitive task (Mandrick et al., 2013).

In the present study, a relationship between brain’s hemodynamic responses and physical performance (under anerobic supramaximal exercise conditions) has been revealed. The participants were divided into two groups by means of PP mentioned as high- and low-performers in order to address the performance relationship. Despite the similar hemodynamic response patterns during pre-exercise session of cognitive task, different hemodynamic response patterns have been observed between groups during post-exercise sessions of cognitive task (Figures 3, 4). As the most striking difference the rise of oxy-Hb and total-Hb levels of HP group have been found statistically higher than those of LP group in central and left PFC areas. These findings may demonstrate that the rise of oxygen consumption and demand in PFC is higher in the HP group, as known as higher anerobic capacity, than the LP group. In this context, Drigny et al. (2014), reported that the brain oxygenation could change with training in obese patients. Also, Khan and Hillman (2014) reviewed the connection between training and its effects on brain oxygenation levels and suggested a relationship between erobic fitness and cognitive processes that can be demonstrated via different fMRI and EEG studies. A similar relationship was described between exercise and cognitive functions by Davenport et al. (2012). In the light of results of the present study, level of PFC oxygenation in physically better performing group during post-exercise cognitive task might be higher than the physically lower performing group during post-exercise cognitive tasks.

The current study also demonstrated a significant correlation between peak power (exercise load) and post-exercise change of hemodynamic responses (Δoxy-Hb, Δdeoxy-Hb, and Δtotal-Hb). This correlation also supports the aforementioned assumptions in which the higher performers have higher PFC oxygenation. This can become a valuable parameter for future studies on human factor by means of physical/cognitive load. Formerly using a different paradigm, our group has demonstrated the centrofrontal shift of the active brain areas due to cognitive load (Bayazıt et al., 2009). In that study, the cognitive load—such as conflict resolution—displayed a physical drive from the posterior areas towards frontal areas with an increased demand on the frontal regions. Similarly, in the current study, the high peak power task is not solely a motor one but also requires a multitude of cognitive activities. In order to cope with the higher motor (and cognitive) load, the brain has to perform increased levels of concentration, attention, coordination, environment monitoring, and reactive and interactive motor control. The frontal areas are the primary areas for these and similar executive and complex skills. Further clarification is needed in this context to describe the effect of physical fitness on brain oxygenation by means of new study designs and larger size groups.

The effects of acute exercise on cognitive functions have been investigated for decades. However, studies demonstrating the mechanisms of physical exercise on brain oxygenation in relation to cognitive performance via fNIRS are very limited and these mechanism are not well understood (Albinet et al., 2014; Drigny et al., 2014; Dupuy et al., 2015). In previous studies, the participants’ performance for decision-making, mental processing speed, selective attention, and reaction time was investigated (Aks, 1998; Arcelin and Brisswalter, 1999; Emery et al., 2001). Moreover, Brisswalter et al. (2002) and Tomporowski (2003) reported that the cognitive performance could be improved by acute exercise. Interestingly, previous studies revealed different findings depending on the type of the exercise, while intense and exhausting exercise causes fatigue (Brisswalter et al., 2002), and light exercise (Varner and Ellis, 1998) causes cognitive performance impairment. Besides, some studies reported a decrease in cognitive functions related with the fatigue caused by the intensity or duration of the exercise (Mehta and Parasuraman, 2014). The physiological basis of this decrement could also be originated from the natural challenge between PFC area related with cognition and motor area related to motion, which causes a hypo-frontality (Dietrich, 2003). The experimental setup of the current study employed a high load but very short (30-sec) anerobic exercise, and such exercise model could cause higher oxygenation responses in PFC similar as erobic exercise models. As mentioned before, the behavioral results displayed only a small but non-significant increase in WA and slight but not significant increase in the CA after the exercise. Our results did not provide a strong support for neither an improvement nor decrement in cognitive scores after acute exercise.

In the related literature, Stroop test was frequently employed as a cognitive task, and generally the improvement of the total test time was accepted as an indicator of improved cognitive functions (Hyodo et al., 2012; Endo et al., 2013). In the present study we employed the 2-Back test as a cognitive task, and evaluated the whole parameters (CA, WA, MA, CART, TRT), but not the total test time. Therefore 2-Back test might not be the right tool as an indicator of the improved cognitive functions. Also the rise of PFC oxygenation could not fully mean an improvement in cognitive function during the post-exercise session. Another plausible explanation might be that the participants had to work harder “neurally” (indicated by increased oxy-Hb levels) to maintain the cognitive performance during post-exercise session.

In another view, some studies reported an improvement of cognitive functions after the moderate exercise (Potter and Keeling, 2005; Coles and Tomporowski, 2008). However, a recent meta-analysis (McMorris et al., 2011) raised an issue. Those authors examined the effect of acute, moderate intensity exercise on working memory tasks and found that speed and accuracy of processing were differentially affected. In particular, the positive effects of acute exercise seem to be disproportionately influential on executive control processes (i.e., planning, coordination, inhibition, mental flexibility, working memory) relative to tasks of recall and alertness (Chang et al., 2012; McMorris and Hale, 2012). N-Back task used in the current study indeed involves working memory, alertness, planning, and inhibition strategies. Therefore a possible explanation could be that there might be an increase in the cognitive (i.e., executive control process) functions due to prolonged total blood flow capacity increase in the brain metabolic capacity (both oxygen and glucose should be increased) but somehow 2-Back test might not be tough enough to elucidate such improvement. Another explanation could be that the increased metabolic activity in brain might be the mean of more neural effort to maintain the performance, not the indicator of cognitive improvement.

The findings of current literature are not sufficient to explain positive effects of exercise intensity on cognitive functioning (Soga et al., 2015). This study employed a short-term high intensity and all-out exercise model. Following this anerobic exercise, 2-Back test was administered and its results showed a non-significant enhancement in cognitive scores. The different effects of anerobic and erobic exercise on cognitive scores could cause the insignificant differences in test results despite the increase in brain oxygenation. Also the variation in cognitive tests that administered could lead such insignificant results. Finally, the administration time of post-exercise tests could affect the cognitive scores. Soga et al. (2015) reported that there is no clear information in literature about cool-down periods following exercise and administration time of cognitive test. In addition, they (Soga et al., 2015) underlined the importance of assessing both time and heart rate variables post-exercise before administering the cognitive tests for future studies.

In the related literature, it has been suggested that the increment in PFC oxygenation has a positive effect on PFC functions which uses erobic exercise (Endo et al., 2013). Our study has no statistically significant behavioral results to demonstrate such positive effects on PFC functioning. Though the current study used anerobic exercise model, the results (hemodynamic responses) are in line with the studies which consists erobic exercise model (Ando et al., 2011; Endo et al., 2013; Byun et al., 2014). The obtained changes in oxygenation levels are compatible with results of previous studies. But no statistical differences were found in cognitive performance parameters, which were positively affected by erobic exercise. The major dividing line between the erobic and anerobic exercise are the duration and intensity parameters. And these erobic definitions are mostly related to the body and muscle metabolism. As far as we know there is no certain study that has specifically displayed the dividing line between erobic and anerobic capacity in regard to brain only. This might be pointing to the fact that the relationship of the body metabolic capacity and the dynamic patterns might be different than of the brain’s.

There are findings demonstrating that cognitive functions, especially following an intense exercise, could be impaired or not augmented. Soga et al. (2015) reported that exercise has different effects on working memory and inhibitory control components. In our study, despite the PFC oxygenation increment, no statistical differences were found between 2-Back test scores. The different effect mechanisms, exercise intensity, and administration time of cognitive tests could be the reason for this insignificancy. In future studies, with an experimental design employing both erobic and anerobic exercises, the hemodynamic and cognitive performance changes can be demonstrated.

Current study may have a considerable limitation. Heart rate may not recover linearly during post exercise (erobic or anerobic), it is likely that cerebral oxygenation, which is regulated by autonomic responses also change drastically from exhaustion to 1 min post recovery to 5 min post recovery. Therefore even though the oxygenation recordings were done post 5 min plus period we may have a bias in the aspect of fluctuations within the 5 min average periods.

## Conclusion

In summary, acute supramaximal exercise increased the oxy-Hb, deoxy-Hb and total-Hb levels during post-exercise session of 2-Back test. This would indicate that the functions of PFC would increase after acute exercise but the comparison between pre- and post-exercise sessions of 2-Back test scores did not point out any significant improvement. These results could not reveal any cognitive performance augmentation despite the increased PFC oxygenation. As aforementioned in the discussion, such post-exercise oxygenation increase in PFC may not be only derived from cognitive performance. To clarify the basis and effects of oxygenation rise further studies is required with an experimental design, which consist the cognitive tasks in all three phases (pre-, during-, post-exercise).

HP group, who has higher PP, outperformed LP group by means of oxy-, deoxy-, and total-Hb changes. Such difference rise a question if the short-term brain oxygenation responses might be affected by physical performance. The current study could provide a methodological approach for human factor studies that would require combining of behavioral and objective methods (fNIRS, etc).

## Author Contributions

The study was planned by CSB, AO, and MO, with experimental design by CSB, CG, EUD, EG, CC. Data was collected and analyzed by CG, EUD, HO, CC. Work was drafted by EUD, CC, EG, and HO. Important intellectual support was given by CSB, AO, MO, EG, and CG throughout the project. All authors prepared the manuscript and approved the final version of the manuscript.

## Funding

This study was funded partly via Dokuz Eylul University, Department of Scientific Research Projects coded by 2012.KB.SAG.081 and 2014.KB.SAG.012.

## Conflict of Interest Statement

The authors declare that the research was conducted in the absence of any commercial or financial relationships that could be construed as a potential conflict of interest.
